# Folate vitamers in the Australian green plum: Through growth and ripening and across locations

**DOI:** 10.3389/fnut.2022.1006393

**Published:** 2022-10-14

**Authors:** Selina Fyfe, Hung Hong, Horst Joachim Schirra, Heather E. Smyth, Yasmina Sultanbawa, Michael Rychlik

**Affiliations:** ^1^Queensland Alliance for Agriculture and Food Innovation, The University of Queensland, Brisbane, QLD, Australia; ^2^Griffith Institute for Drug Discovery, Griffith University, Brisbane, QLD, Australia; ^3^School of Agriculture and Food Science, The University of Queensland, Brisbane, QLD, Australia; ^4^School of Environment and Science, Griffith University, Brisbane, QLD, Australia; ^5^Centre for Advanced Imaging, The University of Queensland, Brisbane, QLD, Australia; ^6^Chair of Analytical Food Chemistry, Technical University of Munich, Freising, Germany

**Keywords:** green plum, *Buchanania obovata*, folate, 5-CH_3_-H_4_folate, 5-methyl-tetrahydrofolate, micronutrients, vitamers, Anacardiaceae

## Abstract

The green plum is a native fruit of Australia that grows on the tree *Buchanania obovata*. This study aimed to confirm the high level of folate in green plums by analyzing a large number of ripe samples from multiple locations and to understand how folate vitamers change as the fruit grows through maturity stages. This study analyzed green plums for five vitamers of folate, H_4_folate, 5-CH_3_-H_4_folate, 5-CHO-H_4_folate, 10-CHO-PteGlu, and PteGlu (folic acid) using a stable isotope dilution assay on a liquid chromatograph mass spectrometer (LC-MS). Green plums were tested from four locations, two harvests and five maturity stages. Another 11 ripe samples, each from different tree clumps from one location, were also tested as were ripe red-colored green plums. The results show the 5-CH_3_-H_4_folate in green plum increases and accumulates in the fruit through development, ripening and senescence. The ripe green plums contain between 82.4 ± 5.5 and 149.4 ± 10.7 μg/100 g Fresh Weight (FW). The red-colored green plums are even higher in folate, with total folate measured as 192.5 ± 7.0 and 293.7 ± 27.4 μg/100 g FW, and further analysis of them is suggested. There is some variation in amounts of folate between fruit from different locations and sets of trees, but all ripe green plums tested are considered good dietary sources of folate.

## Graphical Abstract

Green plum total folate results as fresh weight for five maturity stages of green plum growth and by locations and harvest. 
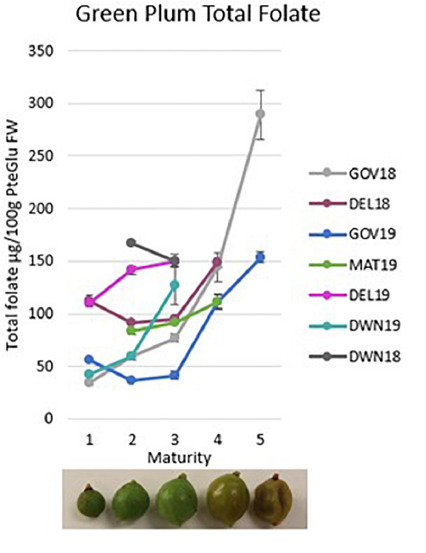

## Introduction

The green plum is a native fruit of Australia that grows on the tree *Buchanania obovata* in the northern parts of the Northern Territory and Western Australia (1). The flesh and seed of the green plums are harvested and eaten by Aboriginal Australians who live in the remote places where it grows (2). It ripens in the hot humid summer of these tropical areas around the time of the first monsoonal rain (3). Green plums are eaten as a raw fruit from the tree (4), when they have been sundried (5) or when the flesh and seed are pounded together into a pulp (6). Green plums are wild harvested and have not yet been commercialized, however, there is interest from Aboriginal communities in Australia to use the green plum as part of sustainable Indigenous bush food businesses.

An initial study of the nutritional properties of the green plum, that was performed on slightly underripe green plums, discovered it was a good dietary source of folate, containing 752.4 μg/100 g Dry Weight (DW) or 161 μg/100 g Fresh Weight (FW) as pteroylmonoglutamic acid equivalents (7). A further study on ripe green plums found the total folate to be 118 μg/100 g FW (8). This amount of folate indicates the green plum is promising as a dietary source of folate, however, further testing of more green plum samples and from fruit across different locations was necessary to be able to confirm that the folates are consistently high.

Folate vitamers, also known as the B9 vitamins, are essential components of metabolism for all humans, plants and animals. Some plants, bacteria and fungi are able to synthesize folates, whereas humans and animals require it from dietary sources (9). Folate is important in human health as its deficiency can lead to neural tube defects in fetuses, megaloblastic anemia, cardiovascular disease, cancer and neurological disorders (10). Folate is such an important vitamin in health that many foods such as breads and cereals are now fortified with folic acid to counter deficiencies in the diets of whole populations (10, 11). In Australia, wheat flour sold for making bread must contain between 2 and 3 mg/kg of folic acid (12). Finding promising key sources of folate in understudied plant foods could help with decreasing micronutrient deficiencies across the world and contribute to future nutritional resilience (13). Underutilized plant foods, including native Australian plant foods, can add biodiversity to food and agriculture to increase food production and increase options for a balanced diet (14).

The amounts of specific nutritional compounds in fruit can vary across locations and harvest years due to differences in climate conditions, soil types and environmental variables (15). The aim of this study was to analyze green plums for folate vitamers from multiple locations and harvest years, to obtain more results on ripe fruit, and to understand how the vitamers change during fruit growth and ripening. For this study, fruit were obtained from four locations in the Northern Territory, three of these locations were sampled for two harvests, and fruit were harvested at all stages of development and divided into a maturity index to obtain folate results for these maturity stages. Five folate vitamers were measured and total folate was calculated as the sum of them. The folate vitamers measured were tetrahydrofolate (H_4_folate), 5-methyltetrahydrofolate (5-CH_3_-H_4_folate), 5-formyltetrahydrofolate (5-CHO-H_4_folate), and 10-formylfolic acid (10-CHO-PteGlu) which are all generally present in fruit and vegetables, and the fifth vitamer measured was pteroylmonoglutamic acid/folic acid (PteGlu). This information will be useful for Aboriginal communities interested in creating sustainable Indigenous bush food businesses and for the food industry.

## Materials and methods

### Samples

**FIGURE 1:**
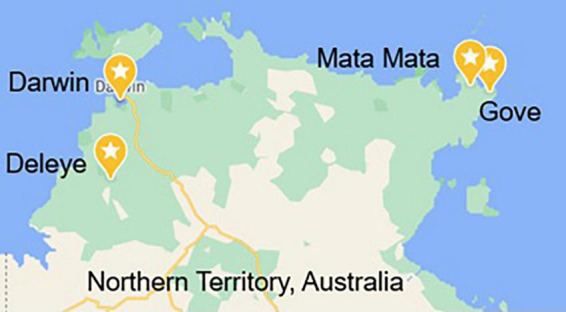
Map of the four locations in the Northern Territory, Australia, that the green plums were harvested from Map data^©^ 2022 Google.

Samples were collected by staff from Gulkula Mining Company Pty Ltd., Arnhem Land Progress Aboriginal Corporation (ALPA), Wild Orchard Kakadu Plum Pty Ltd., by researchers in this study and with the assistance of botanist Greg Leach (Charles Darwin University) and environmental officer Annemarie van Doorn (Gulkula and ALPA) and were obtained under Northern Territory Parks and Wildlife Permit numbers 64749, 64579, and 66293. Samples were obtained from four locations, Gove (GOV), Deleye (DEL), Darwin (DWN), and Mata Mata (MAT) (Figure 1), and from two harvests designated 18 and 19, the year in which that green plum harvest began, as the fruit ripen and are harvested between November and February. GOV and MAT were both from the north-east part of the Northern Territory in East Arnhem Land and DEL and DWN were from the north-west part of the Northern Territory (Figure 1). Fruit from MAT were only obtained in the second harvest year. Green plum samples from GOV, DEL and DWN were identified by the Queensland Herbarium as *Buchanania obovata* and were incorporated into the Herbarium collection as AQ952392, AQ952393, and AQ952402. The accepted name for this species is *Buchanania obovata* Engl. from the family Anacardiaceae (16).

**FIGURE 2:**
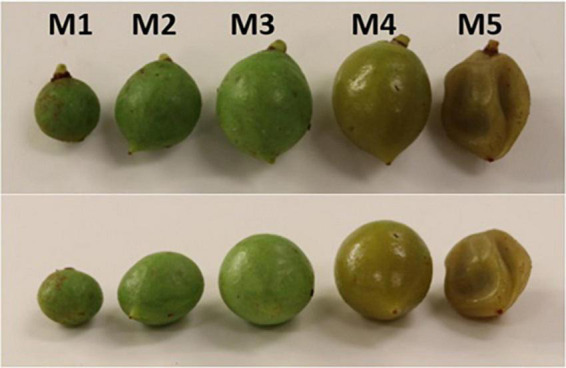
Maturity index of Green Plums from left to right: M1, M2, M3, M4, M5 from two different views.

The fruit were harvested at all stages of maturity and were divided into five maturity stages to make a maturity index designated M1, M2, M3, M4, and M5 (Figure 2), which are described as:

M1: Smallest size, 0–25% in size, hard green, juvenile
M2: Medium size, 25–50% in size, hard green, juvenile
M3: Largest hard green mature fruit
M4: Yellower and softer, skin more translucent, ripe, edible
M5: Overripe or senescence, yellowish, dryer and wrinkled, edible

M1 and M2 are the juvenile fruit, M3 is the mature green fruit, M4 is the ripe fruit and M5 is ripened fruit that have dried in a similar way to how a sultana is dried. From GOV fruit were harvested from 11 separate locations within a 4-h drive and ripe M4 fruit from each of these locations was analyzed.

Red-colored green plums were harvested in both harvest years from GOV and were also analyzed for folate.

Whole fruit were frozen to –20°C within 1 day of collection and remained frozen during transportation to Brisbane and during storage until further processing. The fruit were cut and had the seeds removed by hand, and the flesh and skin of each composite sample was lyophilized in a Christ Gamma 1–16 LSC Freeze Drying Unit (John Morris Scientific, Osterode, Germany), then ground to a fine powder in a ball mill at 30 rotations per second (Lab Mill, Christy and Norris Ltd., Chelmsford, England) and frozen at –20°C until analysis.

### Chemicals

L-ascorbic acid, sodium chloride, sodium hydroxide, disodium hydrogen orthophosphate and potassium dihydrogen orthophosphate were obtained from Chem-Supply (Gillman, SA, Australia). 1,4-dithiothreitol (DTT) was purchased from Roche (Basel, Switzerland). Sodium acetate trihydrate, methanol and acetonitrile were obtained from Merck (Darmstadt, Germany). 2-(N-morpholino)-ethanesulfonic acid (MES) came from Sigma-Aldrich (Steinheim, Germany). Formic acid was from Honeywell (Seelze, Germany). Strata SAX cartridges were from Phenomenex (Aschaffenburg, Germany). Chicken pancreas was from Pel-Freez Biologicals (Rogers, AR, USA) and rat serum was from Bio-Rad (Gladesville, NSW, Australia). The unlabeled folate vitamer reference compounds, pteroylmonoglutamic acid/folic acid (PteGlu), tetrahydrofolate (H_4_folate), 5-methyltetrahydrofolate (5-CH_3_-H_4_folate), 5-formyltetrahydrofolate (5-CHO-H_4_folate), and 10-formylfolic acid (10-CHO-PteGlu) and the ^13^C-labeled isotopological internal standards, ^13^C_5_-PteGlu, ^13^C_5_-H_4_folate, ^13^C_5_-5-CH_3_-H_4_folate, and ^13^C_5_-5-CHO-H_4_folate were from Schircks Laboratories (Bauma, Switzerland).

### Sample and reference preparation

The preparation of the samples, the reference analyses and the isotopological internal standards for the stable isotope dilution assay used to quantify the folate vitamers has previously been described (17, 18). In short, triplicate replicates of 0.06–0.07 g of freeze-dried powder for each sample were weighed into glass tubes. Extraction buffer (10 mL) containing 200 mM MES, ascorbic acid (20 g/L) and DTT (pH 5.0) (1 g/L) was added and were equilibrated. Internal standards of ^13^C_5_-PteGlu, ^13^C_5_-H_4_folate, ^13^C_5_-5-CH_3_-H_4_folate and ^13^C_5_-5-CHO-H_4_folate were then added in amounts adjusted to the expected content of the analytes to fall in the given calibration line. Tubes were boiled at 100°C for 10 min, cooled on ice and enzymes for deconjugation were added as 2 mL chicken pancreas suspension (1 g/L in phosphate buffer and 1% ascorbic acid) and 0.3 mL of rat serum. Glass tubes were then incubated in the dark in a shaking incubator at 37°C for a minimum of 12 h, then boiled to stop the enzyme activity, cooled on ice, mixed with acetonitrile (10 mL) and centrifuged (4,000 rpm, 20 min, 4°C). The supernatant was purified using strong anion-exchange (SAX, quaternary amine, 55 μm, 70 Å, 500 mg/3 mL) and folates were eluted using an elution buffer of 5% sodium chloride, 1.36% sodium acetate, 1% ascorbic acid and 0.1% DTT. Eluate was membrane filtered at 4°C (0.2 μm PP membrane syringe filter, Pall Corporation, Melbourne, Australia), stored in dark glass vials at –35°C and measured within 10 days of extraction.

Unlabeled reference compounds of PteGlu, H_4_folate, 5-CH_3_-H_4_folate, 5-CHO-H_4_folate, and 10-CHO-PteGlu were measured and pre-dissolved in phosphate buffer. H_4_folate, 5-CH_3_-H_4_folate, 5-CHO-H_4_folate and 10-CHO-PteGlu were each mixed with PteGlu and MES extraction buffer and each reference was stored in a dark glass vial until analysis. A response mixture of unlabeled and ^13^C-labeled reference compounds measured and mixed in MES extraction buffer was made up at the same time as the sample extractions and the unlabeled reference compounds, and stored and measured alongside them.

### Instrumental conditions

Instrument conditions were slightly modified from those used in Striegel et al. (17). The analysis was performed on a Shimadzu Liquid Chromatograph Mass Spectrometer (LC-MS) (Shimadzu Corp., Kyoto, Japan) equipped with a Shimadzu 8,060 triple-stage quadrupole mass spectrometer, three Nexera X2 LC-30AC liquid chromatographs, a Nexera X2 SPD-M30A diode array detector, a Nexera X2 C20-20AC prominence column oven, a Nexera X2 SIL-30AC autosampler, and a Raptor ARC-18 column (2.7 μm, 100 × 2.1 mm) (Restek, Bad Homburg, Germany).

The mobile phase used for the binary gradient was a mixture of (A) 0.1% formic acid in distilled water and (B) acetonitrile with 0.1% formic acid and the flow rate was 0.40 mL/min. After 1.5 min the gradient was set to 3% B and raised to 10% B linearly within 3 min. The gradient was held at 10% B for 1.5 min then raised to 15% B over 5 min then to 50% B over 1 min and held for 1 min then reduced to 5% over 1 min and 3% over 4 min. The injection volume used was 5 μL and each injection had a total run time of 18 min. The column effluent was diverted to the mass spectrometer between 2.8 and 11 min and otherwise ran to the waste valve. The MS operated in multiple reaction monitoring mode for MS/MS measurements. The ion source parameters had a heat block temperature of 400°C, dilution line temperature 250°C, interface temperature 300°C, drying gas and heating gas flow were 10 L/min, nebulizing gas flow 2 L/min, column oven temperature was 30°C and collision-induced dissociation gas 270 kPa. Retention time for the folate vitamers were PteGlu 6.4, H_4_folate 3.7, 5-CH_3_-H_4_folate 4.3, 5-CHO-H_4_folate 5.9, and 10-CHO-PteGlu 5.8. Each sample replicate was analyzed on the instrument three times.

### Calculations and statistics

Results were calculated using the measured amount of PteGlu in comparison to the unlabeled reference compounds in the reference solution. These were used in conjunction with the unlabeled and ^13^C-labeled folate vitamers in the response measurement following the method of Striegel et al. (17). Results were calculated using Microsoft Excel v 2014 spreadsheets (Microsoft, Redmond, WA, USA). All results are given as μg/100 g as pteroylmonoglutamic acid equivalents in fresh weight (FW) form unless stated as dry weight (DW). The lower limit of quantification was 0.1 PteGlu in μg/100 g FW. Statistical analysis of the results was done using IBM SPSS software (IBM Australia Ltd., Sydney, NSW, Australia) using analysis of variance (ANOVA) and significantly different results were further tested using Tukey Honestly Significant Difference (HSD) tests.

## Results and discussion

### Folate in green plums through growth and ripening

The folate results for the green plums for the five maturity stages, the four locations and two harvest years are given in Table 1. Results are given for each of the five folate vitamers tested in fresh weight and for the sum of these which is the total folate in fresh weight. Total folate for each sample is also given in dry weight. A strawberry reference sample was analyzed alongside the green plum samples, it was previously determined to be 93.7 ± 6.5 μg/100 g (17), and in the present analysis yielded 87.2 ± 2.6 μg/100 g.

**TABLE 1:** Total folate content and distribution of vitamers in green plums with samples identified by location, harvest year, and maturity stage, results are presented as PteGlu in μg/100 g on fresh weight basis except for far right column which gives total folate on a dry weight basis.

Sample	5-CH3-H4folate	5-CHO-H4folate	10-CHO-PteGlu	H4folate	PteGlu	Total folate	Total folate (dry weight)
Gov 18 M1	24.3 ± 1.7^a^	8.5 ± 0.9^a^	0.8 ± 0.1^b^	0.3 ± 0.4^a,b^	0.3 ± 0.3^a^	34.2 ± 1.9^a^	223.7 ± 12.1^a^
Gov 18 M2	38.0 ± 1.9^a,b^	20.4 ± 1.1^d,e,f^	<LOQ^a^	1.0 ± 0.4^a,b,c,d,e,f^	<LOQ^a^	59.5 ± 3.0^b,c^	428.0 ± 21.3^c^
Gov 18 M3	55.7 ± 3.9^b,c^	20.5 ± 0.6^d,e,f^	<LOQ^a^	0.7 ± 0.0^a,b,c,d^	<LOQ^a^	77.1 ± 4.4^c,d^	478.7 ± 27.6^c,d^
Gov 18 M4	126.1 ± 12.8^g,h,i^	17.9 ± 1.2^c,d,e^	<LOQ^a^	<LOQ^a^	<LOQ^a^	144.0 ± 13.8	475.7 ± 45.4^c,d^
Gov 18 M5	260.4 ± 23.3^j^	26.4 ± 1.8^g^	<LOQ^a^	2.2 ± 1.0^d,e,f^	< LOQ^a^	289.0 ± 23.1^j^	571.9 ± 45.6^e,f^
Del 18 M1	73.6 ± 6.4^c,d^	37.0 ± 0.7^h,i,j^	<LOQ^a^	1.8 ± 1.2^b,c,d,e,f^	<LOQ^a^	112.4 ± 5.4^e,f^	673.2 ± 32.4^g,h^
Del 18 M2	68.0 ± 0.9^c^	23.2 ± 1.3^e,f,g^	<LOQ^a^	0.3 ± 0.3^a,b^	<LOQ^a^	91.6 ± 1.0^d,e^	571.9 ± 6.0^e,f^
Del 18 M3	73.7 ± 1.8^c,d^	20.5 ± 1.3^d,e,f^	<LOQ^a^	0.3 ± 0.4^a,b^	<LOQ^a^	94.5 ± 2.1^d,e^	589.5 ± 13.2^e,f^
Del 18 M4	125.8 ± 5.2^g,h,i^	22.5 ± 0.6^d,e,f,g^	<LOQ^a^	0.3 ± 0.3^a,b^	<LOQ^a^	148.6 ± 4.9^g,h,i^	722.1 ± 23.8^h,i^
Dwn 18 M2	133.7 ± 0.6^h,i^	33.1 ± 1.9^h^	<LOQ^a^	0.6 ± 0.4^a,b,c,d^	<LOQ^a^	167.3 ± 1.7^i^	1063.5 ± 11.1^k^
Dwn 18 M3	114.5 ± 2.9^f,g,h^	34.4 ± 0.9^h,i^	<LOQ^a^	0.7 ± 0.2^a,b,c,d^	0.4 ± 0.7^a^	150.0 ± 1.9^g,h,i^	882.6 ± 11.1^j^
Gov 19 M1	41.4 ± 1.3^a,b^	14.2 ± 1.0^b,c^	<LOQ^a^	0.3 ± 0.1^a,b^	<LOQ^a^	55.9 ± 1.9^a,b,c^	337.4 ± 11.3^b^
Gov 19 M2	25.8 ± 1.2^a^	10.4 ± 0.9^a,b^	0.2 ± 0.3^a^	0.5 ± 0.7^a,b,c,d^	<LOQ^a^	36.9 ± 1.3^a,b^	237.2 ± 8.1^a^
Gov 19 M3	27.0 ± 2.8^a^	14.5 ± 1.0^b,c^	<LOQ^a^	<LOQ^a^	<LOQ^a^	41.5 ± 3.7^a,b^	272.0 ± 24.1^a,b^
Gov 19 M4*	97.0 ± 6.0^e,f^	13.8 ± 1.0^b,c^	<LOQ^a^	0.2 ± 0.3^a,b^	<LOQ^a^	111.1 ± 6.3^e,f^	444.5 ± 25.2^c,d^
Gov 19 M5	139.9 ± 5.8^i^	11.0 ± 0.8^a,b^	<LOQ^a^	2.5 ± 0.6^f^	0.3 ± 0.3^a^	153.7 ± 4.9^h,i^	506.5 ± 16.2^c,d,e^
Del 19 M1	67.6 ± 2.1^c^	41.7 ± 1.9^j^	<LOQ^a^	0.9 ± 0.2^a,b,c,d,e,f^	<LOQ^a^	110.2 ± 3.0^e,f^	522.4 ± 14.2^d,e^
Del 19 M2	100.7 ± 3.5^e,f^	39.1 ± 0.8^i,j^	<LOQ^a^	1.6 ± 0.6^a,b,c,d,e,f^	<LOQ^a^	141.5 ± 3.7^g,h^	711.9 ± 18.5^h,i^
Del 19 M3	109.1 ± 2.9^e,f,g^	39.3 ± 5.1^i,j^	<LOQ^a^	2.1 ± 1.5^c,d,e,f^	<LOQ^a^	150.4 ± 6.6^h,i^	791.0 ± 34.6^i^
Dwn 19 M1	24.2 ± 1.1^a^	17.4 ± 1.5^c,d^	<LOQ^a^	0.5 ± 0.4^a,b,c,d^	<LOQ^a^	42.1 ± 2.3^a,b^	203.0 ± 11.0^a^
Dwn 19 M2	44.0 ± 2.0^a,b^	14.8 ± 1.7^b,c^	<LOQ^a^	0.8 ± 0.3^a,b,c,d,e,f^	<LOQ^a^	59.6 ± 1.0^b,c^	286.5 ± 5.0^a,b^
Dwn 19 M3	99.8 ± 17.2^e,f^	24.9 ± 1.6^f,g^	<LOQ^a^	2.4 ± 0.3^e,f^	<LOQ^a^	127.2 ± 18.5^f,g^	619.1 ± 90.0^f,g^
Mat 19 M2	58.3 ± 1.0^b,c^	23.4 ± 2.8^f,g^	<LOQ^a^	1.6 ± 0.3^a,b,c,d,e,f^	<LOQ^a^	83.4 ± 3.0^d^	500.9 ± 17.8^c,d,e^
Mat 19 M3	68.9 ± 2.7^,c,d^	22.0 ± 2.3^d,e,f,g^	<LOQ^a^	0.6 ± 0.3^a,b,c,d^	0.2 ± 0.2^a^	91.8 ± 0.9^d,e^	581.1 ± 5.4^e,f^
Mat 19 M4	90.7 ± 7.0^d,e^	20.4 ± 0.3^d,e,f^	<LOQ^a^	0.4 ± 0.5^a,b,c^	<LOQ^a^	111.5 ± 7.5^e,f^	446.2 ± 30.0^c,d^

<LOQ is less than the limit of quantification, *Gov 19 M4 result is the average of the 11 results from Table 2, means followed by the same letter within columns were not significantly different (*p* < 0.05).

Of the folate vitamers tested, H_4_folate, 5-CH_3_-H_4_folate, and 5-CHO-H_4_folate are generally present in fruit and vegetables. The results in Table 1 show that 5-CH_3_-H_4_folate is the most abundant folate present in green plums at all maturity stages and in all locations and both harvests. The green plum results show mostly increases in total folate FW in the green plums through growth, maturation, and ripening and each total folate result is mostly comprised of 5-CH_3_-H_4_folate. Table 1 shows that 5-CH_3_-H_4_folate mostly increases in green plums as it matures between M1 and M3 although DWN 18 and GOV 19 show some decreases. There is a marked increase through ripening between M3 and M4 in all samples sets and again through senescence up to M5. The second highest folate vitamer in green plums is 5-CHO-H_4_folate which has a slight decrease in all of the locations and harvest years tested between M3 and M4, through the ripening process, except for DEL 18 which increases slightly. PteGlu, H_4_folate, and 10-CHO-PteGlu are only present in very small amounts in green plums, with levels less than 2.5 μg/100 g in all samples for all of these vitamers. PteGlu is only found naturally in trace quantities (11), as is seen in the green plum. The DW total folate results show there is generally an increase between M2 and M3, except for the DWN 18 samples which show a decrease, however, both these results are at higher levels than the other samples. During ripening, between M3 and M4, there is an increase in the total folate in the DEL 18 and GOV 19 samples, a very slight decrease in the GOV 18 and a decrease in the MAT 19. There is an increase in amount of DW folate vitamers from M4 to M5 that occurs during the drying of the ripe fruit.

When comparing the fresh weight results of green plums with those of other fruits, folates in papaya fruit have an increase in 5-CH_3_-H_4_folate through development and into the ripening stages and then a decrease as it finishes ripening (19). An analysis of six different cultivars of tomato through ripening, from unripe through to full ripe, did not show any clear trend of increasing or decreasing 5-CH_3_-H_4_folate content through four stages of ripening (20). Tomato fruit that are mature green have been measured as having total folate at 21.93 ± 1.17 μg/100 g FW which was slightly higher than the total folate measured on ripe red tomato’s at 18.05 ± 2.01 μg/100 g FW (21). Two other studies also show higher levels of folate in mature green tomatoes then in red ripe ones (22) and one of these showed a decrease from green mature to red ripe tomatoes in 5-CH_3_-H_4_folate content on three different cultivars (23). Analysis of folates in the kernel of waxy maize through development showed decreasing total folate through four stages of development (24).

Folates are synthesized by plants and the folate vitamers interconvert between themselves as part of the metabolism process (25–27). The biosynthesis pathways of folate in plants have been characterized and well-studied (28). This current analysis adds to plant metabolism knowledge by giving an understanding of the regulation of folate in the green plum. Folates are only synthesized in the mitochondria of plant cells where the pterin and *p*-aminobenzoic acid (*p*ABA) moieties join into a H_4_folate molecule, however, they are also found throughout the cell in the plastids, cytosol and vacuoles (26, 29). Folate vitamers, including four of the vitamers in this study, are important in metabolic pathways involving one-carbon transfers. It is the reduced folate forms, including H_4_folate and 5-CH_3_-H_4_folate, which transfer the one-carbon units and are the core of one-carbon metabolism providing single carbon units for anabolic reactions (27). These one-carbon substituents are primarily methyl or formyl groups that exist on the N^5^ or N^10^ positions but can also be methylene, methenyl or formimino groups (11, 30). One-carbon transfers are important in amino acid catabolism, 10-CHO-H_4_folate is required for purine ring biosynthesis and contributes to formylmethionyl-tRNA synthesis (27) and 5,10-CH_2_-H_4_folate is required for synthesis of thymidine which is needed for DNA synthesis (30). 5-CH_3_-H_4_folate acts as a methyl donor during the synthesis of methionine from homocysteine and methionine is converted to S-adenosyl methionine (SAM) (10, 30) and in plants SAM is involved in methyltransferase reactions and the synthesis of biotin and ethylene (27). Folates are essential for the development of plants because of their role in DNA synthesis (26). Folates are present in the green plum from the juvenile stage and at mostly increasing levels as the cells grow and divide enabling DNA synthesis to occur. The 5-CHO-H_4_folate has some accumulation in green plums with higher levels in those from DEL and DWN then from GOV and MAT but without major trends in increasing or decreasing seen. The 5-CH_3_-H_4_folate is the vitamer that is at the highest level and which accumulates the most throughout fruit development. This indicates it is produced at faster rates then the rate it is being used. Further studies on the genetics of green plums, including genomics, transcriptomics, and proteomics, could provide more information about folate biosynthesis and metabolism in this fruit.

The original study on the green plum was from underripe fruit from the same region as the DWN samples and the total folate was 161 μg/100 g FW (7). This is slightly higher than the 18 M3 result from DWN which was 150.0 μg/100 g FW. Ripe, M4, samples were not available for testing for either harvest year from this location, however, these results do confirm the original published green plum folate analysis. Variation in metabolite content in fruit may be caused by many different factors, and the differences seen between location and harvest year in this study could be due to environmental differences such as soil type, rainfall or sunlight, genetic differences, or normal variation between trees.

### Folate in ripe green plums

The green plums are eaten when they are ripe (M4) or when they have been semi-dried in the sun (M5). Analysis of the folate vitamers of ripe green plums was done on fruit from 11 different sets of trees in the GOV region during the second harvest to determine the natural variation that occurs in one region. These results are presented in Table 2 and are a comparison of the natural variation in a location.

**TABLE 2:** Total folate content and distribution of vitamers in ripe green plums from different locations around Gove in East Arnhem Land, calculated as PteGlu in μg/100 g on fresh weight basis except for the total folate dry weight column.

Sample	5-CH3-H4folate	5-CHO-H4folate	10-CHO-PteGlu	H4folate	PteGlu	Total folate	Total folate (dry weight)
Gov 19 M4 S1	74.5 ± 4.0^a^	12.6 ± 1.2^c,d^	<LOQ^a^	0.1 ± 0.2^a^	<LOQ^a^	87.2 ± 5.0^a^	348.8 ± 20.0^c^
Gov 19 M4 S2	129.1 ± 10.3^e^	19.5 ± 0.7^e^	<LOQ^a^	0.8 ± 0.5^a^	<LOQ^a^	149.4 ± 10.7^c^	597.8 ± 42.7^a^
Gov 19 M4 S3	92.6 ± 3.8^a,b,c^	19.3 ± 1.7^e^	<LOQ^a^	<LOQ^a^	<LOQ^a^	111.9 ± 5.5^b^	447.8 ± 22.1^a,b^
Gov 19 M4 S4	79.0 ± 9.3^a,b^	6.4 ± 0.6^a^	<LOQ^a^	<LOQ^a^	<LOQ^a^	85.4 ± 9.5^a^	341.8 ± 37.9^a,b^
Gov 19 M4 S5	96.2 ± 6.3^b,c^	14.9 ± 0.6^d^	<LOQ^a^	0.2 ± 0.2^a^	<LOQ^a^	111.3 ± 6.5^b^	445.3 ± 26.0^b^
Gov 19 M4 S6	118.9 ± 7.5^d,e^	18.5 ± 1.0^e^	<LOQ^a^	0.2 ± 0.3^a^	<LOQ^a^	137.6 ± 6.5^c^	550.7 ± 25.9^c^
Gov 19 M4 S7	100.1 ± 1.2^c,d^	14.4 ± 1.5^d^	<LOQ^a^	<LOQ^a^	<LOQ^a^	114.5 ± 1.9^b^	458.1 ± 7.7^c^
Gov 19 M4 S8	129.6 ± 11.4^e^	10.9 ± 0.6^b,c^	<LOQ^a^	0.1 ± 0.1^a^	<LOQ^a^	140.6 ± 11.1^c^	562.5 ± 44.2^a^
Gov 19 M4 S9	83.7 ± 2.1^a,b,c^	15.0 ± 1.6^d^	<LOQ^a^	0.7 ± 0.6^a^	<LOQ^a^	99.4 ± 3.0^a,b^	397.7 ± 12.0^a^
Gov 19 M4 S10	73.1 ± 5.9^a^	9.1 ± 0.3^a,b^	<LOQ^a^	0.2 ± 0.3^a^	<LOQ^a^	82.4 ± 5.5^a^	329.9 ± 22.1^b^
Gov 19 M4 S11	90.7 ± 4.2^a,b,c^	11.2 ± 0.9^b,c^	<LOQ^a^	0.4 ± 0.6^a^	<LOQ^a^	102.2 ± 4.0^a,b^	409.2 ± 16.0^b^
*Average:*	*97.0* ± *6.0*	*13.8* ± *1.0*	<*LOQ*	*0.2* ± *0.3*	<*LOQ*	*111.1* ± *6.3*	*444.5* ± *25.2*

<LOQ is less than the limit of quantification; means followed by the same letter within columns were not significantly different (*p* < 0.05).

The total folate results of the ripe, M4, fruit from GOV were between 82.4 ± 5.5 and 149.4 ± 10.7 μg/100 g FW and all of the M4 samples from other locations also fall in this same range. The total folate on ripe green plums previously published was also from fruit from this location and was found to be 118 μg/100 g FW (8) which is near the middle of the range in this study. Tables 1, 2 together show the variation between all of the ripe fruit in this study from all of the locations and harvest years. The two highest vitamers in the ripe fruit are 5-CH_3_-H_4_folate and 5-CHO-H_4_folate. This variation in ripe fruit is similar to the variation seen in strawberry cultivars which have been measured between 93.0 ± 5.3 and 153 ± 5.0 μg/100 g FW using the same method (17). Despite the variation seen in ripe green plums and strawberries, all of the results are still high when compared to other fruits. Green plums and strawberries both have higher folate levels than many other fruits including grape, kiwi, mandarin, watermelon, pineapple (31) and tomatoes (4 to 60 μg/100 g FW) (22). Green plum has higher folate than mango which is also in the family Anacardiaceae (55.8 ± 0.73 to 74.5 ± 2.09 μg/100 g FW) (32). It is higher than the tropical fruits guavas (43.1 ± 5.16–103 ± 4.32 μg/100 g FW), papayas (61.6 ± 3.01–90.7 ± 1.24 μg/100 g FW), jackfruit (52.9 ± 2.61–83.6 ± 5.50 μg/100 g FW), dragon fruit (36.0 ± 0.53 μg/100g FW), prickly pear (23.8 ± 0.44 μg/100 g FW), tamarillo (16.4 ± 0.60 μg/100 g FW) and tamarind (11.4 ± 0.70 μg/100g FW) (32). Despite this, the green plums folate levels are still within the natural range and are below those of the durian, which has been measured as high as 440 ± 7.89 μg/100g FW (18) and passion fruit (136 ± 21.7–271 ± 3.64 μg/100 g FW) (32).

Compared with other Australian native fruit, the green plum has higher levels of folate than most of those that have been analyzed. The green plum range in dry weight for ripe, M4, fruit is between 329.9 ± 22.1 and 597.8 ± 42.7 μg/100 g DW for the GOV and MAT fruit and the DEL 18 M4 fruit was measured at 722.1 ± 23.8 μg/100 g DW. The green plums are higher in folate than the quandong (120 μg/100 g DW), the Kakadu plum, riberry, and lemon aspen (all 110 μg/100 g DW) and Davidson’s plum (34–40 μg/100 g DW). The Australian desert lime had levels within the green plum range (420 μg/100 g DW) (33). People in remote Aboriginal communities generally have poor quality diets with many nutrients coming from fortified processed foods (34). The remote locations are difficult to transport fresh fruit and vegetables too, however, bush foods, the fruit and vegetables that are native to these areas, are still eaten by these Aboriginal communities (2). The results show that the green plum is a good dietary source of folate for these remote communities in Australia.

The edible M5 semi-dried fruit had even higher FW and DW results then the ripe M4 fruit. The first harvest year result was 289.0 ± 23.1 and the second 153.7 ± 4.9 μg/100 g FW which is higher than folate levels in dried sultana’s, currants, apricots and prunes which have been measured between 13.8 and 83.8 μg/100 g FW (35). These high levels in the M5 fruit also indicate that studies on the degradation of folates in green plums at different storage temperatures should be done to understand their stability, as these vitamers have increased as the fruit has dried.

In humans folate deficiency has been linked to megaloblastic anemia as folate and vitamin B12 are closely linked in metabolite pathways (30). Folate is important for maintaining normal cell growth and division and for the repair, methylation and synthesis of DNA (10, 36). 5-CH_3_-H_4_folate is the most abundant form of folate that circulates in the human body and it can cross the intestinal barrier and move into the blood stream (10) or be transported into peripheral tissues (37). Folic acid, PteGlu, is used as a supplement in case of and to prevent folate deficiency (10, 11). Bread is commonly fortified with folate, however, the bioaccessibility of the folate in different bread types ranges from 31 to 120% (38). In strawberry fruit the bioaccessibility of folate vitamers has been found to be between 90 and 95% when subjected to *in vitro* gastrointestinal digestion and colonic fermentation (39). A human pilot study of folate bioavailability of strawberries found a recovery from plasma of 96.2% (40). Biofortified tomatoes analyzed for natural folate bioavailability using a murine model has been found to be comparable to the bioavailability of synthetic 5-CH_3_-H_4_folate and more than PteGlu (41). 5-CH_3_-H_4_folate has been found to have advantages over synthetic folic acid in human health as it is absorbed even when gastrointestinal pH has been altered, it is bioavailable even when there are metabolic defects, reduces potential to mask symptoms of B12 deficiency and reduces interactions with other drugs (37, 42). Unmetabolised folic acid from exposure to synthetic folic acid *in utero* at higher concentration in babies at birth has been associated with the development of food allergies and food sensitization (43). Therefore, to find fruit with naturally high levels of folate is important for diet and health. The dry weight results of ripe green plums show that as a freeze-dried powder it is very high in folate and future uses of green plum as a nutraceutical or as a natural source of folate, especially of 5-CH_3_-H_4_folate, for fortification of foods should be explored. Further studies on the bioaccessibility of folate vitamers in green plums is suggested. The sensory qualities of green plum shows it has well-liked and unique flavor profile of *sweet*, *tart* and *stewed apple* and has potential to be used as a future flavor (8). This analysis confirms that the folate levels in green plums are consistently high across locations, and this, combined with its desirable sensory qualities, suggest it could be used in novel future food products.

### Folate in red-colored green plums

**FIGURE 3:**
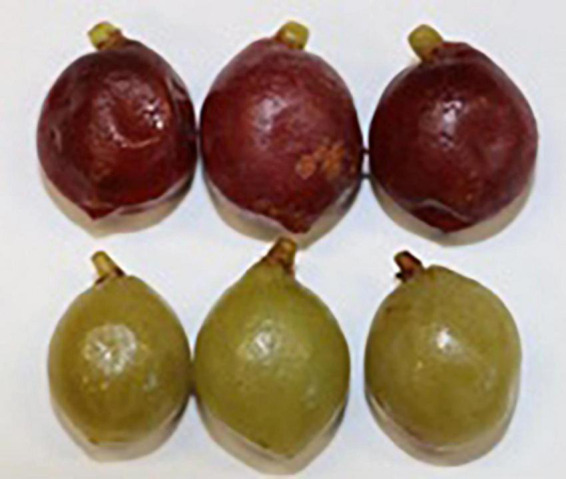
Red-colored **(top row)** and green-colored **(bottom row)** green plums from GOV location.

Two samples of red-colored green plums from the GOV location were also tested for folate content. Figure 3 shows representative examples of the ripe red-colored green plums in the top row and the ripe green-colored green plums in the bottom row for comparison.

The folate results of the red-colored green plums, in Table 3, show they are even higher in folate then the green-colored green plums in both fresh weight and dry weight. Both harvest years have higher levels of total folate and 5-CH_3_-H_4_folate than all of the ripe green-colored green plums that were measured. The amount of 5-CHO-H4folate and H4folate are similar to the highest levels in the green-colored ripe fruit. The red-colored fruit also have less than the limit of quantification for 10-CHO-PteGlu and PteGlu, just as all of the ripe green-colored green plums did. Overall, the red-colored green plums are very high in folate and further analysis of red-colored green plums for both its folate vitamers and other nutritional properties is recommended.

**TABLE 3:** Total folate content and distribution of vitamers in ripe (M4) red-colored green plums from East Arnhem Land, calculated as PteGlu in μg/100 g on fresh weight basis except for total folate dry weight column.

	5-CH3-H_4_folate	5-CHO-H4folate	10-CHO-PteGlu	H4folate	PteGlu	Total folate	Total folate (dry weight)
Gov 18 red	273.4 ± 25.5	19.7 ± 1.7	<LOQ	0.6 ± 0.4	<LOQ	293.7 ± 27.4	929.6 ± 86.9
Gov 19 red	170.6 ± 6.9	21.3 ± 1.3	<LOQ	0.5 ± 0.4	<LOQ	192.5 ± 7.0	609.7 ± 22.0

<LOQ is less than the limit of quantification.

## Conclusion

This study has shown that green plums are high in folate and are a good natural dietary source of it. The green plum 5-CH_3_-H_4_folate increases and accumulates in the fruit through development, ripening and senescence. There is some variation between green plums from different locations and sets of trees but all ripe samples are considered good folate sources. The red-colored green plums are even higher in folate than the green-colored green plums and further analysis of them is warranted. Folates are important for plant metabolism and for human metabolism and this study gives insight into folate vitamers in green plums as they grow and ripen, from different locations and as a dietary source.

## Data availability statement

The original contributions presented in this study are included in the article/supplementary material, further inquiries can be directed to the corresponding author.

## Author contributions

SF, YS, and MR conceptualized the manuscript. SF and HH performed the analysis. SF wrote the manuscript. HJS, HES, YS, and MR supervised the project. SF, HH, HJS, HES, YS, and MR edited the manuscript. All authors contributed to the article and approved the submitted version.
